# A *Ralstonia solanacearum* effector RipAD perturbs aquaporin NbPIP oligomeric status to suppress plant immunity

**DOI:** 10.1111/tpj.71111

**Published:** 2026-10-08

**Authors:** Bo Nian, Sai Yuan, Yijie Liu, Ying Zhang, Pengyue Li, Yang Lin, Jiatao Xie, Jiasen Cheng, Yanping Fu, Xiao Yu, Daohong Jiang, Bo Li

**Affiliations:** ^1^ National Key Laboratory of Agricultural Microbiology Huazhong Agricultural University Wuhan Hubei 430070 China; ^2^ Hubei Hongshan Laboratory Wuhan Hubei 430070 China; ^3^ The Provincial Key Lab of Plant Pathology of Hubei Province College of Plant Science and Technology, Huazhong Agricultural University Wuhan Hubei 430070 China

**Keywords:** bacterial wilt, type III effector, virulence, aquaporin, reactive oxygen species

## Abstract

Bacterial wilt caused by *Ralstonia solanacearum* is a devastating disease that affects a wide range of crops, including tobacco. The pathogen promotes infection by secreting type III effector (T3E) proteins into host plants to facilitate pathogenicity; however, the molecular mechanisms for many of these effectors remain elusive. Here, we characterize RipAD, a conserved T3E that significantly contributes to *R. solanacearum* virulence in *Nicotiana tabacum* and *Nicotiana benthamiana*. We demonstrate that RipAD dampens early immune responses, including pathogen‐associated molecular pattern‐triggered reactive oxygen species (ROS) bursts and MAPK activation. This immunosuppressive function depends on the dual localization of RipAD to the plasma membrane and the nucleus. Mechanistically, RipAD physically associates with the N‐terminal domains of the plasma membrane intrinsic proteins NbPIP2;3 and NbPIP2;4. We show that these aquaporins act as positive regulators of ROS accumulation and plant immunity. Crucially, RipAD binding is associated with reduced homo‐ and hetero‐oligomerization of NbPIP2;3 and NbPIP2;4, thereby compromising their stability and function. Furthermore, silencing *NbPIP2;3* and *NbPIP2;4* increases tobacco susceptibility and rescues the virulence defect of a RipAD deletion mutant. These findings reveal a novel virulence strategy wherein a bacterial effector targets the structural integrity of host aquaporin complexes to block ROS signaling and suppress immunity.

## Significance Statement

Our study identifies RipAD, a *Ralstonia solanacearum* effector indispensable for virulence, as a suppressor of early immune signaling. Mechanistically, RipAD binds to and destabilizes the oligomeric complexes of *Nicotiana benthamiana* aquaporins NbPIP2;3 and NbPIP2;4, thereby dampening H_2_O_2_ accumulation and immune activation, uncovering a novel virulence paradigm in which bacterial pathogens target host water channels to subvert a core node of plant defense.

## INTRODUCTION

The *Ralstonia solanacearum* species complex (RSSC) comprises a diverse group of globally distributed bacterial strains that inflict devastating bacterial wilt on over 450 plant species, including major economic crops such as tomato (*Solanum lycopersicum*), potato (*Solanum tuberosum*), and tobacco (*Nicotiana tabacum*) (Jiang et al., 2017; Mansfield et al., 2012). Following invasion through plant roots, the pathogen colonizes stem xylem vessels, subsequently spreads rapidly to aerial tissues, and eventually induces wilting and death of the host plant. Multiple factors underpin the virulence of *R. solanacearum* during infection (Genin & Denny, 2012), with key virulence determinants encompassing cell wall‐degrading enzymes, extracellular nucleases, exopolysaccharides (EPS), and type III effectors (T3Es) (Peeters et al., 2013).

The type III secretion system (T3SS) and its associated effectors are the principal virulence determinants driving *R. solanacearum* pathogenicity (Landry et al., 2020). The RSSC strains encode a vast repertoire of 50–75 T3Es, designated *R. solanacearum* injected proteins (Rips). Delivered via the Hrp T3SS, these effectors are injected into plant cells to subvert host immunity and facilitate infection (Vailleau & Genin, 2023). Recent studies have elucidated molecular mechanisms by which specific Rips modulate plant defense responses. For instance, RipP2 specifically acetylates WRKY transcription factors to suppress the expression of pattern‐triggered immunity (PTI)‐related genes (Sarris et al., 2015). RipAW and RipAR suppress reactive oxygen species (ROS) production and the expression of defense genes (Nakano et al., 2017). The core T3E RipAB disrupts salicylic acid signaling by targeting TGA transcription factors (Qi et al., 2022). Understanding the complex interplay between effectors and host targets is critical for elucidating resistance mechanisms and devising strategies to mitigate bacterial wilt disease.

To defend against pathogens, plants utilize plasma membrane (PM)‐localized pattern recognition receptors (PRRs) to detect pathogen‐associated molecular patterns (PAMPs) and initiate PTI (Jones & Dangl, 2006; Ngou et al., 2022). This recognition activates downstream immune signaling, including calcium influx, MAPKs phosphorylation, transcriptional reprogramming, and notably, a rapid ROS burst (Kadota et al., 2014; Li et al., 2014; Thor et al., 2020). The apoplastic ROS burst is primarily driven by PM‐localized NADPH oxidases (RBOHs), which transfer electrons from cytosolic NADPH to extracellular oxygen (O_2_) to generate superoxide anions. These anions are subsequently dismutated into hydrogen peroxide (H_2_O_2_) (Liu & He, 2016; Torres, 2010). Crucially, to function as a second messenger and activate cytosolic defense pathways, this apoplastic H_2_O_2_ must traverse the PM (Maurel et al., 2015). This transmembrane influx is facilitated by specific members of the aquaporin (AQP) family (Sies et al., 2017), positioning them as essential gatekeepers of ROS‐mediated immunity.

PM intrinsic proteins (PIPs) constitute the largest subfamily of AQP that mediate transmembrane transport of water and other small molecules (e.g., glycerol, urea) (Afzal et al., 2016). Based on sequence similarity, PIPs are divided into two subgroups: PIP1 and PIP2, which differ primarily in the lengths of their N‐ and C‐terminal tails (Yaneff et al., 2015). PIP2 proteins typically serve as efficient water channels, whereas PIP1s often exhibit lower water permeability but facilitate solute transport (Otto et al., 2010; Postaire et al., 2010; Tornroth‐Horsefield et al., 2006). PIPs are increasingly recognized as gatekeepers of plant immunity (Li et al., 2020). In *Arabidopsis thaliana*, AtPIP1;4 mediates the transmembrane influx of apoplastic H_2_O_2_ to activate MAPKs signaling and callose deposition during *Pst* DC3000 infection. Similarly, the wheat TaPIP2;10 facilitates H_2_O_2_ transport to coordinate defense against various fungal pathogens and aphids, a process regulated by site‐specific phosphorylation (Lu et al., 2022). However, the regulatory functions of PIPs in plant innate immunity remain to be explored.

An increasing number of studies have shown that PIPs can be exploited by diverse plant pathogens to facilitate infection. In soybean (*Glycine max*), GmPIP2;33 regulates H_2_O_2_ transport and is targeted by the *Phytophthora sojae* effector CRN78 to subvert plant defense. It is noteworthy that PIPs assemble into homo‐ or heterotetramers, and this oligomerization is crucial for their water‐channel function (de Groot & Grubmuller, 2001; Frick et al., 2013; Mathai & Agre, 1999; Tornroth‐Horsefield et al., 2006). However, SCMV disrupts the oligomerization of ScPIP2;4 in sugarcane (*Saccharum* spp. hybrid), affecting its PM localization and H_2_O_2_ transport capacity, which weakens immune defense (Zhang et al., 2024). It remains unknown whether bacterial pathogens have evolved specific effectors that dismantle PIP oligomeric structures to suppress plant immunity and, if so, what molecular mechanisms underlie this process.

In this study, we identified the effector RipAD from *R. solanacearum* strain NB‐1, which was isolated from wilted tobacco plants. We demonstrate that RipAD is required for virulence and physically associates with select AQPs, including NbPIP2;3 and NbPIP2;4. This interaction impairs the oligomeric architecture of these PIPs, thereby attenuating NbPIP‐mediated H_2_O_2_ transport and suppressing host PTI to facilitate pathogen colonization. These findings reveal a novel virulence strategy employed by *R. solanacearum*, in which RipAD manipulates the structural stability of AQPs and subverts defense pathways.

## RESULTS

### RipAD contributes to *R. solanacearum* virulence in tobacco

To assess the biological function of RipAD in the NB‐1 strain, originally isolated from a tobacco bacterial wilt sample, we generated three independent RipAD deletion mutants (*Δripad*) via homologous recombination (Figure S1A). Furthermore, complementation strains (*ComRipAD*) were constructed by introducing a native promoter‐driven RipAD‐HA cassette into *Δripad* and target protein expression was verified by immunoblotting (Figure S1B). To evaluate basic biological characterization, *Δripad* and *ComRipAD* strains were cultured on both solid and liquid media. Colony size, color, and morphology of mutant strains were comparable to the wild type (WT) strain on triphenyl tetrazolium chloride (TTC) plates (Figure S1C). The growth curves of *Δripad*, *ComRipAD*, and WT strains showed no significant difference (Figure S1D). Furthermore, the pathogenicity‐related biofilm formation, EPS secretion, and cellulose production were similar across all strains (Figure S1E–G). These results indicate that the effector RipAD is dispensable for growth and basic biological traits in *R. solanacearum* strain NB‐1.

To explore the contribution of RipAD to bacterial virulence, we inoculated *N. tabacum cv. SR1* plants with NB‐1, *Δripad* mutant, and complementation strains *ComRipAD* using the soil‐soaking method. The *Δripad* mutant displayed reduced virulence and a relatively lower disease index than the WT strain, while the *ComRipAD* strain restored virulence to WT levels (Figure 1A,B). Consistent with this, *N. tabacum* plants infected with the *Δripad* mutant showed higher survival rates than those inoculated with the WT strain (Figure 1C). We further quantified bacterial populations in *N. tabacum* stems at 5 days post‐inoculation (dpi). The bacterial titer of the *Δripad* mutant had a 1.5‐log reduction in colony‐forming units (CFU) per gram of stem tissue compared to the WT, whereas the population of the *ComRipAD* reached WT level (Figure 1D).

**Figure 1:**
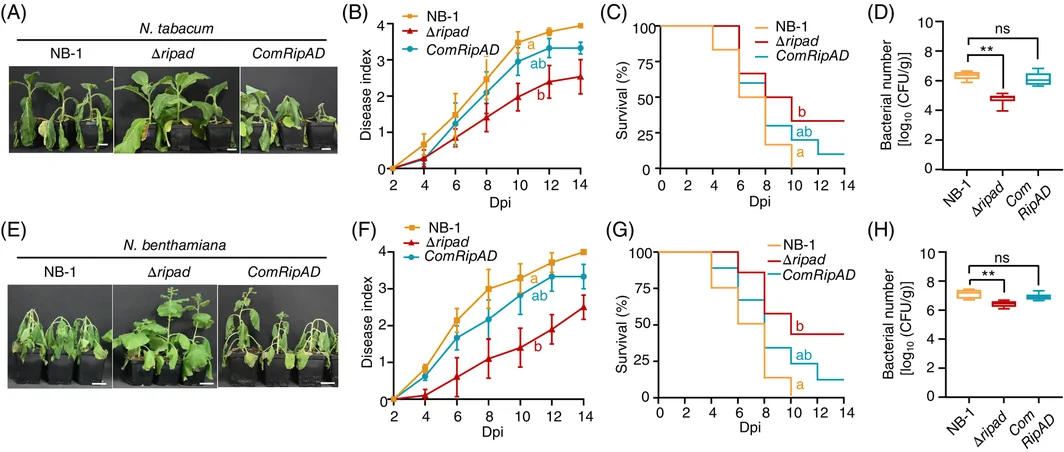
RipAD is required for the virulence of *Ralstonia solanacearum* NB‐1 in tobacco. (A) RipAD is essential for the induction of bacterial wilt symptoms in *Nicotiana tabacum*. Plants were inoculated with wild‐type NB‐1, *Δripad* mutant, or ComRipAD strains via soil drenching. Representative photographs were taken at 10 dpi. Scale bars, 5 cm. (B, C) RipAD contributes to rapid disease progression and host mortality in *N. tabacum*. The disease index (B) and survival rate (C) were recorded every 2 days from 0 to 14 dpi. (D) RipAD promotes efficient bacterial colonization in *N. tabacum* stems. Bacterial titers were quantified at 5 dpi. (E) RipAD is required for full symptom development in *Nicotiana benthamiana*. Soil‐drenching inoculation and photography were performed as in (A). (F, G) RipAD confers full virulence to *R. solanacearum* in *N. benthamiana*. Disease progression (F) and survival rates (G) were monitored as in (B) and (C). (H) RipAD supports bacterial multiplication in *N. benthamiana* stems. Bacterial populations were quantified at 5 dpi. Data in (B), (C), (F), and (G) are presented as mean ± SD (*n* = 8). Statistical significance was determined using one‐way anova based on the area under the disease progression curve (AUDPC); different letters indicate significant differences (*P* < 0.05). Data in (D) and (H) are presented as box plots (*n* = 8), where the center line represents the median and whiskers indicate the minimum and maximum values. Asterisks indicate significant differences determined by Student's two‐tailed *t*‐test (**P < 0.01; ns, not significant).

To further confirm the role of RipAD in *R. solanacearum* virulence, we conducted similar pathogenicity assays on *Nicotiana benthamiana*. Consistent with the results in *N. tabacum*, the *Δripad* mutant caused significantly milder wilt symptoms compared to the WT strain at 7 dpi, and the complementation strain *ComRipAD* fully restored virulence (Figure 1E–G). The disease index of *Δripad*‐inoculated *N. benthamiana* plants was approximately 60% lower than that of WT‐inoculated plants, while *ComRipAD*‐inoculated plants showed a disease index comparable to WT (Figure 1F). Additionally, bacterial population counting in *N. benthamiana* stems at 5 dpi revealed significantly lower bacterial populations than in WT‐inoculated plants, and this defect was rescued in the *ComRipAD* strain (Figure 1H). These results collectively demonstrate that RipAD is a critical virulence factor contributing to *R. solanacearum* NB‐1 pathogenicity in tobacco plants.

### RipAD suppresses multiple branches of immune responses in tobacco

To investigate the biological function of RipAD during *R. solanacearum* infection, we confirmed the transient expression of RipAD protein fused with a green fluorescent protein (GFP) tag (RipAD‐GFP) in *N. benthamiana* leaves (Figure 2A). We then evaluated the impact of RipAD on multiple layers of PTI responses. The results showed that the apoplastic ROS burst triggered by *R. solanacearum* cold shock protein peptide (csp22) or the bacterial flagellin epitope (flg22) was significantly suppressed in leaves expressing RipAD‐GFP, exhibiting an approximately 50% reduction in total ROS accumulation compared to the GFP control (Figure 2B,C). Consistent with the inhibition of ROS accumulation, the activation of MAPKs was markedly attenuated in RipAD‐expressing leaves following stimulation with csp22 or flg22 (Figure 2D,E).

**Figure 2:**
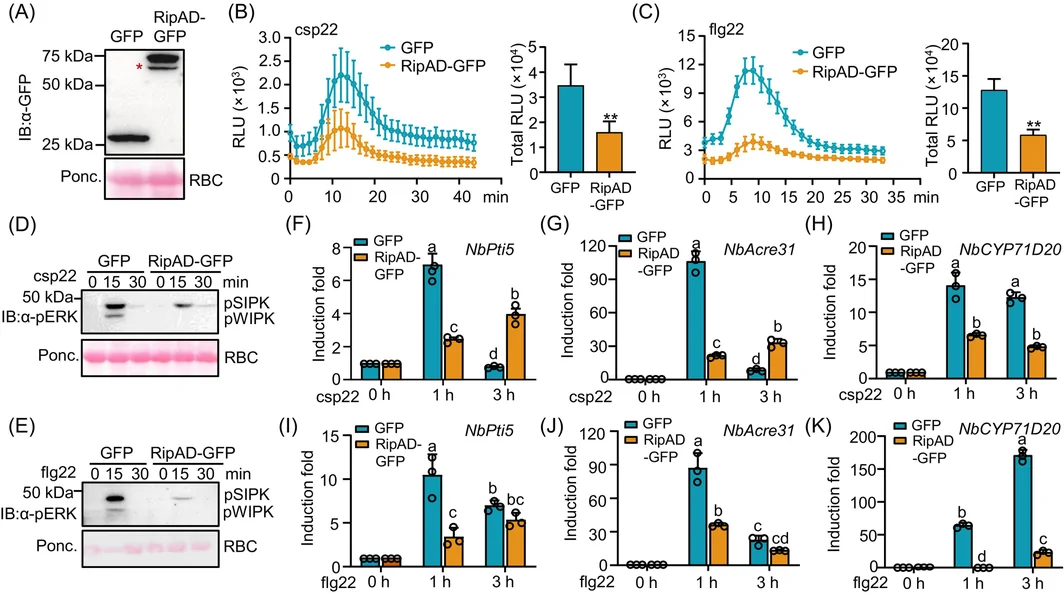
RipAD suppresses plant pattern‐triggered immunity (PTI) immune responses in *Nicotiana benthamiana*. (A) Confirmation of RipAD‐GFP protein expression in *N. benthamiana*. Leaves transiently expressing free green fluorescent protein (GFP) or RipAD‐GFP via agroinfiltration for 2 days were subjected to immunoblot analysis using an anti‐GFP antibody. The red asterisk indicates the specific band for RipAD‐GFP. Ponceau S staining of Rubisco (RBC) serves as a protein loading control. (B, C) RipAD inhibits pattern‐induced reactive oxygen species (ROS) bursts. *Nicotiana benthamiana* leaves transiently expressing GFP or RipAD‐GFP for 2 days were treated with 100 nM csp22 (B) or 100 nM flg22 (C). ROS production dynamics were continuously monitored over time (left panels), and the total ROS accumulation was quantified (right panels) as relative light units (RLU). Total ROS production data are presented as mean ± SE (*n* = 10); asterisks indicate significant differences determined by Student's two‐tailed *t*‐test (**P < 0.01). (D, E) RipAD attenuates MAPKs cascade activation triggered by 100 nM csp22 and 100 nM flg22. Leaves expressing GFP or RipAD‐GFP were treated with 100 nM csp22 (D) or 100 nM flg22 (E) for 0, 15, and 30 min. Activated MAPKs (pSIPK and pWIPK) were detected by immunoblotting using an anti‐pERK antibody. Ponceau S staining of RBC indicates equal protein loading. (F–K) RipAD represses the transcriptional induction of PTI marker genes. Leaves expressing GFP or RipAD‐GFP were treated with 100 nM csp22 (F–H) or 100 nM flg22 (I–K) for 0, 1, and 3 h. The relative expression levels of the PTI marker genes *NbPti5* (F, I), *NbAcre31* (G, J), and *NbCYP71D20* (H, K) were determined by RT‐qPCR and normalized to the internal control *NbEF1α*. Data are shown as mean ± SD (*n* = 3) with individual data points displayed; different letters denote statistically significant differences according to one‐way anova (*P* < 0.05).

Furthermore, we monitored the transcriptional induction of three well‐established PTI‐marker genes: the ERF‐family transcription factor *Pathogenesis‐related protein Pti5 ortholog* (*NbPti5*), the F‐box protein‐encoding gene *Avr9/Cf‐9 rapidly elicited protein 31* (*NbAcre31*), and the phytoalexin biosynthesis‐related gene *Cytochrome P450 71D20* (*NbCYP71D20*) (Heese et al., 2007; Zhong et al., 2018). Reverse transcription‐quantitative polymerase chain reaction (RT‐qPCR) analysis revealed that the expression of these immune‐related genes was strongly induced in the GFP control at 1 and 3 h post csp22 or flg22 treatment. However, this induction was significantly suppressed by RipAD, resulting in a threefold to fivefold reduction in transcript levels compared to the GFP control (Figure 2F–K). Collectively, these results demonstrate that RipAD functions as a potent suppressor of early plant immune signaling.

### Dual localization at the PM and nucleus is essential for RipAD‐mediated immune suppression

Subcellular localization is often intrinsically linked to the biological function of bacterial effectors. RipAD encodes a 310‐amino acid (aa) protein that lacks well‐characterized functional domains and possesses an unknown biochemical function. Phylogenetic analysis revealed that RipAD homologs are widely distributed across various bacterial species, including *Paracidovorax*, *Rhizobium*, *Xanthomonas*, and *Ralstonia* phylotypes I–IV, most of which are annotated as T3Es with uncharacterized functions (Figure S2A). Multiple sequence alignment showed high variability at the N terminus, whereas a highly conserved region was identified in the C terminus, suggesting the presence of a potentially critical functional domain (Figure S2B).

Sequence analysis further identified a potential nuclear localization signal (NLS) and a putative palmitoylation site at cysteine 80 (C80) in the N‐terminal region, which may mediate PM association (Figure S3A). To investigate the subcellular distribution of RipAD, we generated the palmitoylation‐deficient variant RipAD^C80A^‐GFP (*C80A*‐GFP) and the nucleus localization‐deficient variant RipAD^nls^‐GFP (*nls*‐GFP) via site‐directed mutagenesis. Confocal microscopy showed that the WT RipAD‐GFP fusion protein localizes to both the PM and the nucleus in *N. benthamiana* leaves (Figure 3A; Figure S3B). In contrast, the *C80A*‐GFP mutant exhibited impaired and discontinuous PM signal, while the *nls*‐GFP variant showed abolished nuclear localization. To further validate the subcellular localization of RipAD, we performed co‐localization assays using hypersensitive induced reaction 4 (HIR4)‐mCherry as a PM marker and H2B‐mCherry as a nuclear marker (Liu et al., 2024). RipAD‐GFP strongly co‐localized with HIR4‐mCherry at the PM (Figure S3C), and co‐expression with H2B‐mCherry revealed overlapping nuclear signals (Figure S3D). Consistently, confocal laser scanning microscopy showed that the *C80A*‐GFP mutation markedly reduced PM localization, while mutation of the NLS abolished nuclear localization, confirming that C80 and the NLS are essential for their respective localizations.

**Figure 3:**
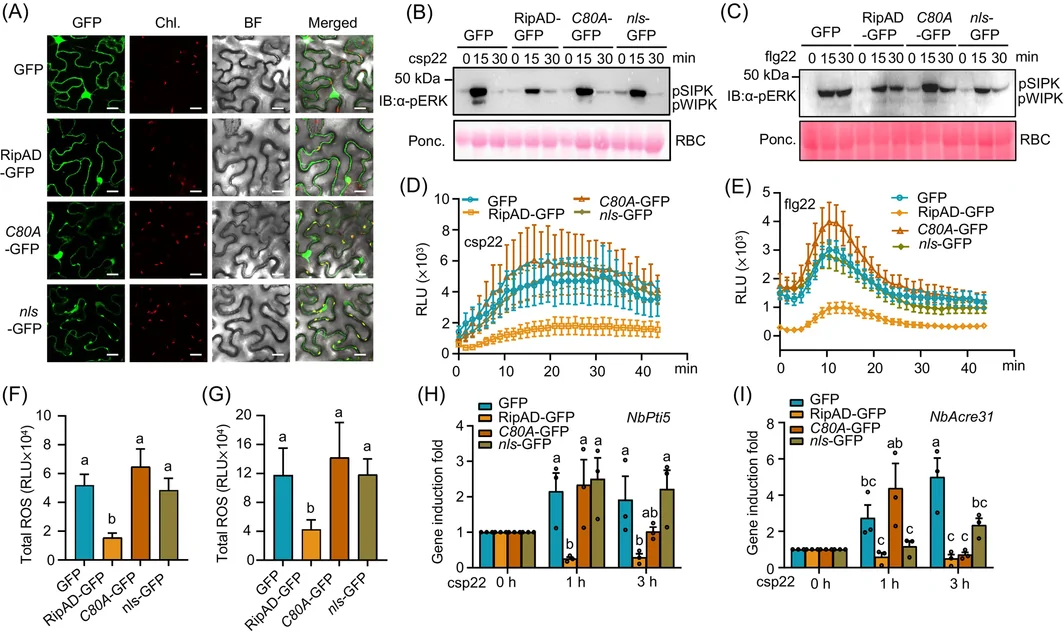
Plasma membrane (PM) and nuclear localizations are essential for the pattern‐triggered immunity (PTI)‐suppressing activity of RipAD. (A) RipAD localizes to both the PM and the nucleus. Confocal microscopy images of *Nicotiana benthamiana* epidermal cells transiently expressing free green fluorescent protein (GFP), wild‐type RipAD‐GFP, the PM‐deficient mutant C*80A*‐GFP, or the nucleus localization deficient mutant *nls*‐GFP. Images were acquired at 2 days post‐infiltration. BF, bright field; Merge, overlay of GFP, chlorophyll autofluorescence (Chl.), and bright field channels. Scale bars, 25 μm. (B, C) PM and nuclear localizations are required for RipAD to suppress MAPK activation induced by csp22 (B) or flg22 (C). Leaves expressing GFP, RipAD‐GFP, *C80A*‐GFP, or *nls*‐GFP proteins were treated with 100 nM csp22 or 100 nM flg22 for 0, 15, and 30 min. Phosphorylated MAPKs were detected by immunoblotting with an anti‐pERK antibody. Ponceau S staining of RBC serves as a loading control. (D–G) Mislocalization of RipAD abolishes its ability to inhibit reactive oxygen species (ROS) burst induced by csp22 (D, F) or flg22 (E, G). ROS dynamics triggered by 100 nM csp22 or 100 nM flg22 were monitored over time in leaf disks expressing the indicated proteins. Total ROS production was quantified for the csp22 (F) and flg22 (G) treatments. Data are presented as mean ± SE (*n* = 10). (H, I) RipAD requires dual localization to repress expression of *NbPti5* and *NbAcre31* induced by csp22. Leaf disks expressing the indicated proteins were treated with 100 nM csp22 for 0, 1, and 3 h and harvested for RT‐qPCR analysis. The expression of *NbPti5* and *NbAcre31* was normalized to that of *NbEF1α*, and values represent mean ± SD (*n* = 3). Different letters indicate statistically significant differences according to one‐way anova (*P* < 0.05). All experiments were repeated three times with similar results.

We next examined whether PM and nuclear localization are required for RipAD‐mediated immune suppression. Unlike the WT form, neither *C80A*‐GFP nor *nls*‐GFP could inhibit the activation of MAPKs induced by csp22 or flg22 (Figure 3B,C). Similarly, the ROS burst triggered by these PAMPs was significantly suppressed by WT RipAD‐GFP, but not by the localization‐deficient variants (Figure 3D–G). Furthermore, both *C80A‐GFP* and *nls*‐GFP lost the ability to restrain the expression of *NbPti5* and *NbAcre31*, the PTI‐related marker genes (Figure 3H,I; Figure S3E,F). Collectively, these results demonstrate that both PM and nuclear localization are indispensable for the ability of RipAD to subvert plant immune responses.

### RipAD physically associates with PIPs in *N. benthamiana*

To identify the host targets of RipAD in tobacco, we conducted liquid chromatography–tandem mass spectrometry (LC‐MS/MS) following biotin‐based proximity labeling (TurboID) and co‐immunoprecipitation (Co‐IP), which yielded over 500 potential candidates. Given the dual localization of RipAD, we prioritized proteins situated at the PM or in the nucleus that exhibited log_2_FoldChange >2 compared to the empty vector (EV) control. Among these candidates, several AQPs were consistently enriched with high peptide intensities (Figure 4A; Figure S4A; Table S1). These included four members of the PIP family, designated as NbPIP2;3, NbPIP2;4, NbPIP2;9, and NbPIP1;3, which belong to distinct phylogenetic subclades (Figure S4A,B).

**Figure 4:**
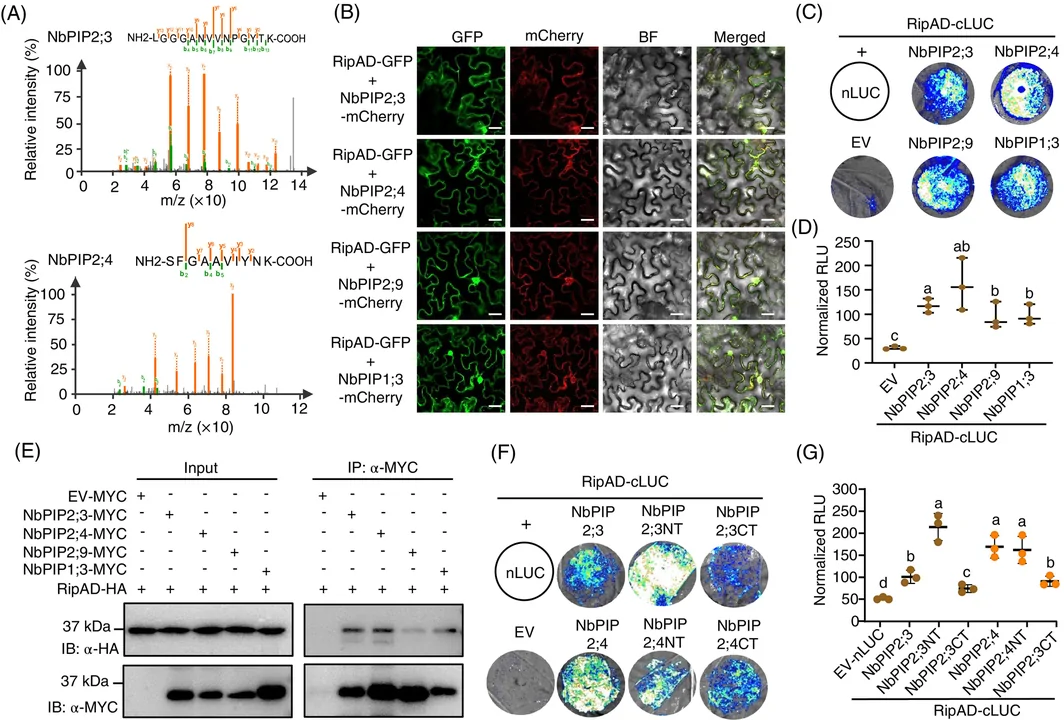
RipAD physically interacts with specific plasma membrane (PM) intrinsic proteins (PIPs) in *Nicotiana benthamiana*. (A) RipAD associates with NbPIP2;3 and NbPIP2;4 *in vivo*. Representative liquid chromatography–tandem mass spectrometry (LC‐MS/MS) spectra of unique peptides identifying NbPIP2;3 and NbPIP2;4. These proteins were identified from the intersection of co‐immunoprecipitation (Co‐IP) (using RipAD‐GFP) and TurboID‐mediated proximity labeling (using RipAD‐TurboID‐HA) assays in *N. benthamiana*. (B) RipAD co‐localizes with NbPIPs at the PM. Confocal microscopy images showing the co‐expression of RipAD‐GFP with NbPIP2;3‐mCherry, NbPIP2;4‐mCherry, NbPIP2;9‐mCherry, or NbPIP1;3‐mCherry in *N. benthamiana* leaves. Images were acquired at 48 hpi. BF, bright field; Merge, overlay of green fluorescent protein (GFP), mCherry, and BF channels. Scale bars, 25 μm. (C, D) RipAD interacts with NbPIPs in split‐luciferase complementation (split‐LUC) assays. (C) Representative images of *N. benthamiana* leaves co‐expressing RipAD‐cLUC and the indicated NbPIP‐nLUC constructs. (D) Quantification of the relative luciferase activity. Luminescence was measured 5 min after substrate addition. (E) RipAD forms a stable complex with NbPIPs *in planta*. Co‐immunoprecipitation (Co‐IP) analysis was performed using *N. benthamiana* leaves co‐expressing RipAD‐HA and the indicated NbPIP‐MYC constructs. Total protein extracts (Input) were immunoprecipitated with anti‐MYC beads, and the presence of RipAD‐HA was detected by immunoblotting. (F, G) RipAD predominantly binds the N‐terminal cytosolic domain of NbPIP2;3 and NbPIP2;4. (F) Split‐LUC assays were performed using RipAD‐cLUC and either the N‐terminal (NT) or C‐terminal (CT) domains of NbPIP2;3 or NbPIP2;4 fused to nLUC. (G) Quantification of the relative luciferase activity for the domain interactions shown in (F). Data in (D) and (G) are presented as mean ± SD (*n* = 3). Different letters denote statistically significant differences according to one‐way anova followed by Tukey's test (*P* < 0.05).

To validate these interactions, we first examined the subcellular distribution of RipAD and the candidate PIPs. Confocal microscopy revealed that RipAD‐GFP co‐localized with mCherry‐tagged NbPIP2;3, NbPIP2;4, and NbPIP2;9 at the PM of *N. benthamiana* epidermal cells (Figure 4B). The physical association was further confirmed using a firefly split‐luciferase complementation (split‐LUC) assay, where co‐expression of RipAD fused to the C‐terminal fragment of luciferase (RipAD‐cLUC) with the four PIPs fused to the N‐terminal fragment of luciferase (nLUC) produced robust bioluminescence signals (Figure 4C,D). Consistent with these findings, *in planta* Co‐IP assays demonstrated that HA‐tagged RipAD (RipAD‐HA) specifically co‐precipitated with all four MYC‐tagged PIPs (PIPs‐MYC; Figure 4E).

To delineate the RipAD‐interacting regions of NbPIP2;3 and NbPIP2;4, we focused on their cytosolic N‐ and C‐terminal regions, as PIPs are transmembrane proteins. Previous studies have identified a highly conserved cysteine residue in extracellular loop A of PIP2 proteins, which forms an inter‐monomeric disulfide bridge and contributes to higher order oligomer assembly (Jozefkowicz et al., 2013). We therefore generated N‐terminal and C‐terminal truncation constructs of NbPIP2;3 and NbPIP2;4; designated TN2‐front and TN2‐rear, respectively; and tested their interactions with RipAD using split‐LUC assays (Figure S4C). Split‐LUC assays indicated that RipAD‐cLUC interacted with both the N‐terminal and C‐terminal fragments of NbPIP2;3 and NbPIP2;4 but showed markedly stronger interactions with the N‐terminal fragment. Full‐length NbPIP2;3 and NbPIP2;4 were included as positive controls for comparison (Figure 4F,G). Furthermore, structural modeling using AlphaFold Server predicted that the binding interface is primarily located within the first 84 amino acids of the N‐terminal region (Figure S4D). Collectively, these results demonstrate that RipAD physically interacts with host PIPs, and this association is predominantly mediated by the N‐terminal domains of the AQPs.

### RipAD perturbs the oligomeric status of NbPIP2;3 and NbPIP2;4

Functional AQP complexes typically assemble into tetramers through the homo‐ or hetero‐oligomerization of individual monomers, which is a structural prerequisite for their stability and channel activity (Barone et al., 1998; Berny et al., 2016). To explore how the effector RipAD modulates the molecular function of host PIPs, we first assessed the baseline homo‐oligomerization of candidate PIPs using split‐LUC assays. The results indicated that NbPIP2;3, NbPIP2;4, and NbPIP2;9 form homodimers in *N. benthamiana* leaves, whereas NbPIP1;3 does not (Figure S5A).

We subsequently investigated whether the physical association with RipAD alters these molecular assemblies. Upon co‐expression with the RipAD protein fused with a hemagglutinin tag (RipAD‐HA), the bioluminescence signals generated by the homodimerization of NbPIP2;3‐nLUC/cLUC and NbPIP2;4‐nLUC/cLUC diminished in a dose‐dependent manner, dropping by nearly 60% at the highest effector concentration (Figure 5A,B). This inhibitory effect was further confirmed by the quantification of relative luciferase activity. In contrast, the homodimerization of NbPIP2;9 remained unaffected even with escalating levels of RipAD (Figure S5B). Furthermore, we analyzed the impact of RipAD on heterodimer formation. Consistent with the observations for homodimers, the heterodimerization between NbPIP2;3 and NbPIP2;4, as well as between NbPIP2;4 and NbPIP2;9, was significantly suppressed by RipAD in a dose‐dependent fashion (Figure 5C,D). Notably, RipAD did not disrupt the interaction between NbPIP2;3 and NbPIP2;9 (Figure S5C).

**Figure 5:**
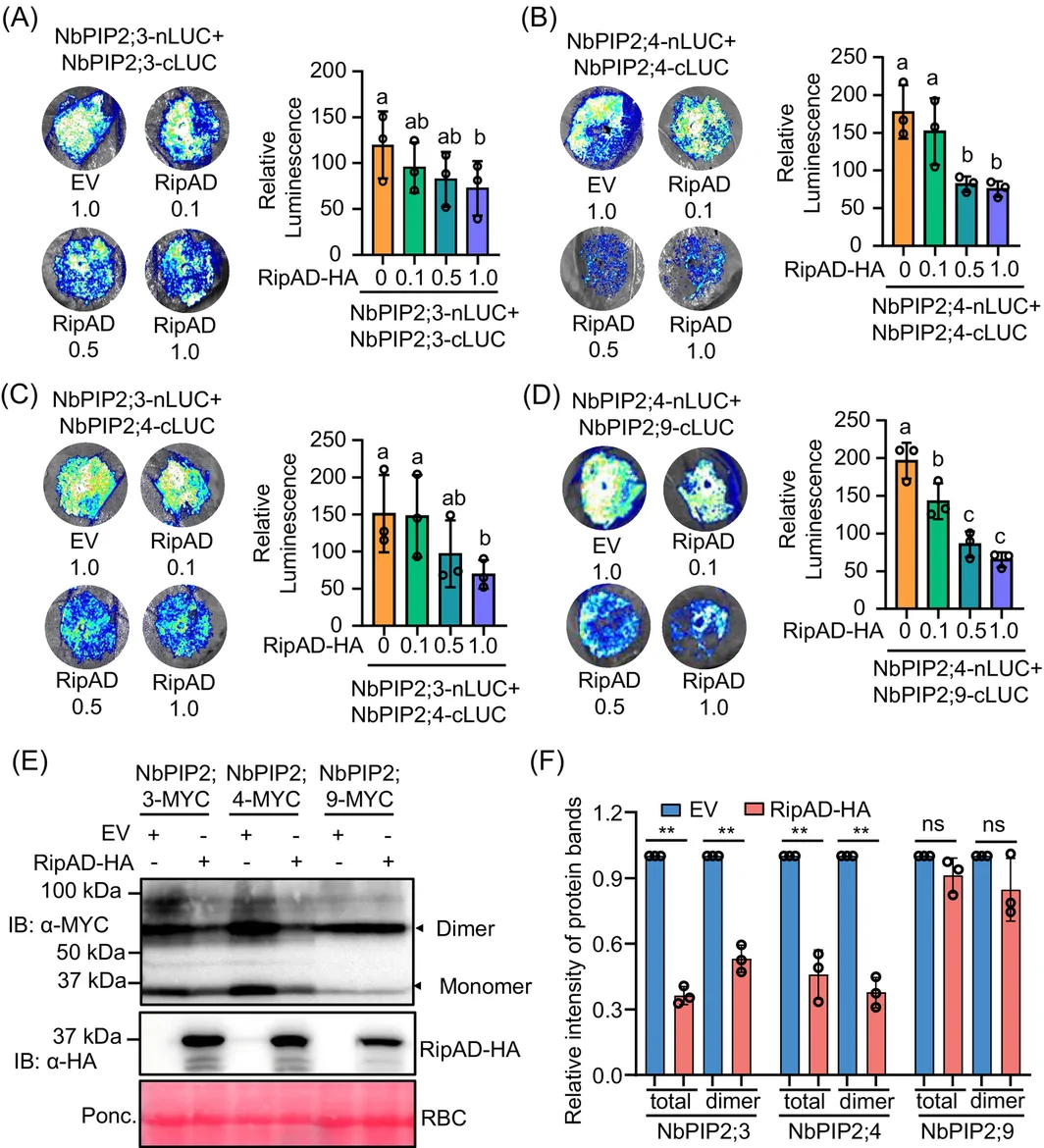
RipAD interferes with the oligomerization of NbPIP2 aquaporins. (A–D) RipAD disrupts the homo‐ and hetero‐dimerization of NbPIP2s in a dose‐dependent manner. *Nicotiana benthamiana* leaves were co‐infiltrated with *Agrobacterium* strains carrying the indicated nLUC and cLUC constructs, along with increasing concentration of the RipAD‐HA strain (OD_600_: 0, 0.1, 0.5, 1.0). Representative images of luciferase luminescence are shown in the left, and quantification of relative luminescence intensity is shown in the right. Data are presented as mean ± SD (*n* = 4); different letters denote statistically significant differences according to one‐way anova followed by Tukey's test (*P* < 0.05). (E, F) RipAD reduces the accumulation of NbPIP2;3 and NbPIP2;4 dimers *in vivo*. Total proteins were extracted from leaves co‐expressing the indicated NbPIP2‐MYC constructs with either empty vector (EV) or RipAD‐HA. Specific bands corresponding to monomers and dimers are indicated by arrowheads. (F) Quantitative analysis of NbPIP2 dimerization and total NbPIP2 protein abundance in (E). The corresponding dimer and total band intensities were quantified and normalized to the respective EV controls. Data are presented as means ± SD (*n* = 3); asterisks indicate significant differences determined by Student's *t*‐test (**P < 0.01; ns, not significant).

To independently verify whether these observations were associated with changes in NbPIP2 oligomeric status and protein abundance, we performed immunoblotting using protein extracts from *N. benthamiana* expressing MYC‐tagged PIPs (PIPs‐MYC) in combination with either an EV control or RipAD‐HA. We observed a significant reduction in the intensities of NbPIP2;3 and NbPIP2;4 dimer bands as well as total NbPIP2 protein abundance in the presence of RipAD compared to the EV control, with quantitative analysis showing a 47 and 63% decrease in dimer abundance, respectively (Figure 5E,F). Notably, NbPIP2;3 and NbPIP2;4 protein levels decreased progressively with increasing RipAD expression (Figure S5D). Collectively, these data demonstrate that RipAD specifically reduces NbPIP2 oligomerization and the protein abundance of NbPIP2;3 and NbPIP2;4 proteins, thereby subverting their physiological roles in plant defense.

### NbPIP2;3 and NbPIP2;4 function as positive regulators of tobacco resistance against bacterial wilt

To investigate the biological significance of the interaction between RipAD and these PIPs, we performed an *Agrobacterium*‐mediated virus‐induced gene silencing (VIGS) assay in *N. benthamiana* to concurrently silence *NbPIP2;3* and *NbPIP2;4*. RT‐qPCR confirmed a marked downregulation of both target genes 3 weeks post‐VIGS compared to the GFP‐silenced control plants (Figure S6A,B). Upon treatment with csp22 or flg22, the ROS burst was significantly diminished in the *NbPIP2;3/2;4*‐silenced plants relative to the controls (Figure 6A). Similarly, the silenced plants exhibited attenuated MAPK activation following flg22 stimulation (Figure 6B). Furthermore, the transcriptional induction of the PTI‐marker genes *NbPti5* and *NbCYP71D20* was remarkably reduced in the silenced plants (Figure 6C,D). These data indicate that NbPIP2;3 and NbPIP2;4 are essential for the activation of csp22‐ and flg22‐triggered PTI in *N. benthamiana*.

**Figure 6:**
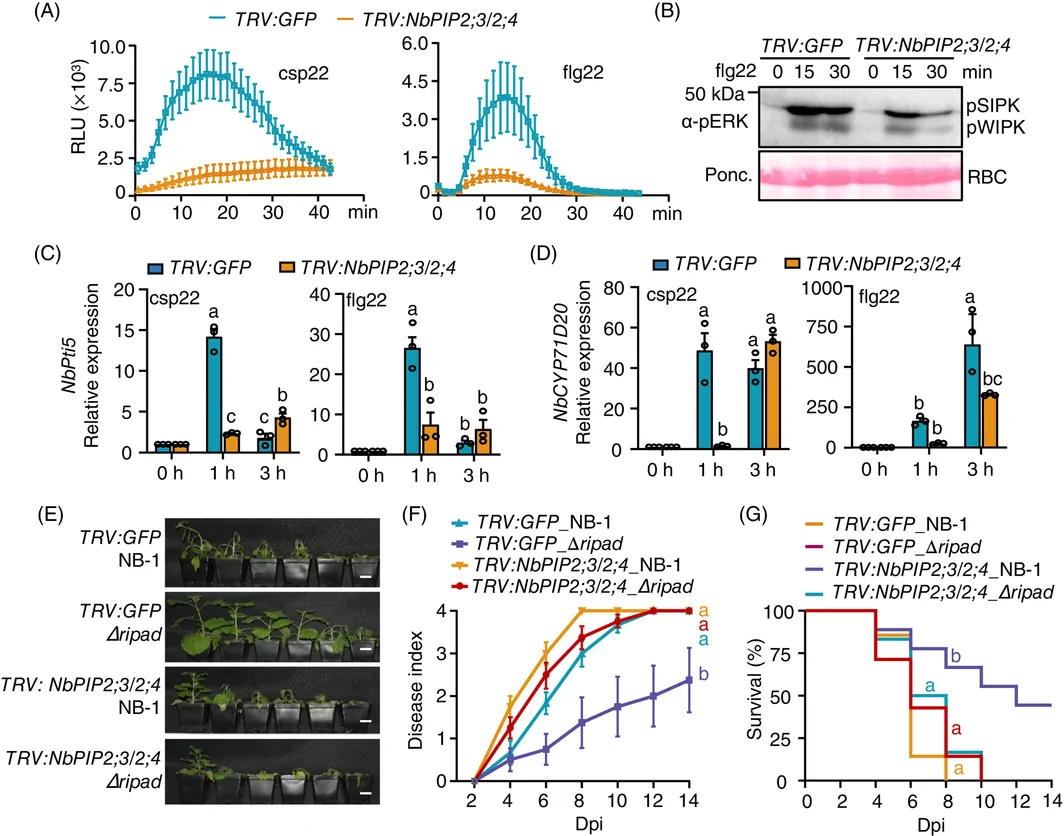
RipAD inhibits *Nicotiana benthamiana* NbPIP2;3/NbPIP2;4 module‐mediated bacterial wilt resistance. (A) NbPIP2;3 and NbPIP2;4 are essential for pathogen‐associated molecular pattern (PAMP)‐triggered reactive oxygen species (ROS) bursts. *NbPIP2;3* and *NbPIP2;4* were cosilenced in *N. benthamiana* using virus‐induced gene silencing (VIGS). Leaf disks from *TRV:GFP* (control) and *TRV:NbPIP2;3/2;4* plants were treated with 100 nM csp22 or 100 nM flg22. Data are presented as mean ± SE (*n* = 10). (B) Silencing of *NbPIP2;3* and *NbPIP2;4* compromises flg22‐induced MAPKs activation. Total proteins were extracted from VIGS plants treated with 100 nM flg22 for the indicated times. Phosphorylated MAPKs were detected by immunoblotting with an anti‐pERK antibody. (C, D) Knockdown of *NbPIP2;3* and *NbPIP2;4* attenuates the expression of pattern‐triggered immunity (PTI) marker genes. Transcript levels of *NbPti5* (C) and *NbCYP71D20* (D) were determined by RT‐qPCR in VIGS plants treated with csp22 or flg22 for 0, 1, and 3 h. Data are normalized to *NbEF1α* and presented as mean ± SD (*n* = 3). (E–G) Silencing of *NbPIP2;3* and *NbPIP2;4* restores the virulence of the *Δripad* mutant. (E) Disease symptoms of *TRV:GFP* and *TRV:NbPIP2;3/2;4* plants inoculated with NB‐1 or *Δripad* strains via soil drenching. Photographs were taken at 10 dpi. Scale bars, 5 cm. (F) Disease progression curves showing that the attenuated virulence of *Δripad* is fully rescued in *NbPIP2;3/2;4*‐silenced plants, reaching levels comparable to the wild‐type NB‐1. (G) Survival rates of plants from the assay in (F). Data in (F) represent the mean disease index ± SE (*n* = 8). Statistical significance was determined using one‐way anova based on the area under the disease progression curve (AUDPC); different letters indicate significant differences (*P* < 0.05).

We further evaluated the resistance of *NbPIP2;3/2;4*‐silenced plants against *R. solanacearum* infection. Following inoculation with the WT strain NB‐1, the silenced plants displayed accelerated and more severe bacterial wilt symptoms compared to the control group, with disease indices reaching nearly 4.0 (complete wilt) by 8 dpi, whereas the control plants remained at a moderate index of approximately 2.5 (Figure 6E–G). Notably, while the *Δripad* deletion mutant exhibited reduced virulence in control plants, it caused intense and rapid wilting in the *NbPIP2;3/2;4*‐silenced lines, characterized by higher disease indices, decreased survival rates, and increased bacterial proliferation (Figure 6E–G). Collectively, these results verify that NbPIP2;3 and NbPIP2;4 are indispensable for resistance against *R. solanacearum* NB‐1; therefore, RipAD strategically targets these PIPs to facilitate infection.

### RipAD suppresses host ROS accumulation and NbPIP2‐mediated H_2_O_2_ transport

Given that PIP AQPs have been implicated in transmembrane H_2_O_2_ transport and ROS‐mediated immune signaling, we next investigated whether RipAD affects NbPIP2‐mediated H_2_O_2_ accumulation during infection. We first performed an *in planta* H_2_O_2_ accumulation assay. 3,3′‐Diaminobenzidine (DAB) staining showed that tobacco roots inoculated with the *Δripad* mutant accumulated significantly higher levels of H_2_O_2_ than those inoculated with the WT strain NB‐1 (Figure 7A,B). Transgenic tobacco lines expressing RipAD under the constitutive 35S promoter were also generated (Figure S6C). Conversely, RipAD‐overexpressing tobacco plants exhibited markedly reduced H_2_O_2_ accumulation after NB‐1 infection compared with WT SR1 plants (Figure 7A,B). These results indicate that RipAD plays an important role in suppressing host ROS accumulation during *R. solanacearum* infection.

**Figure 7:**
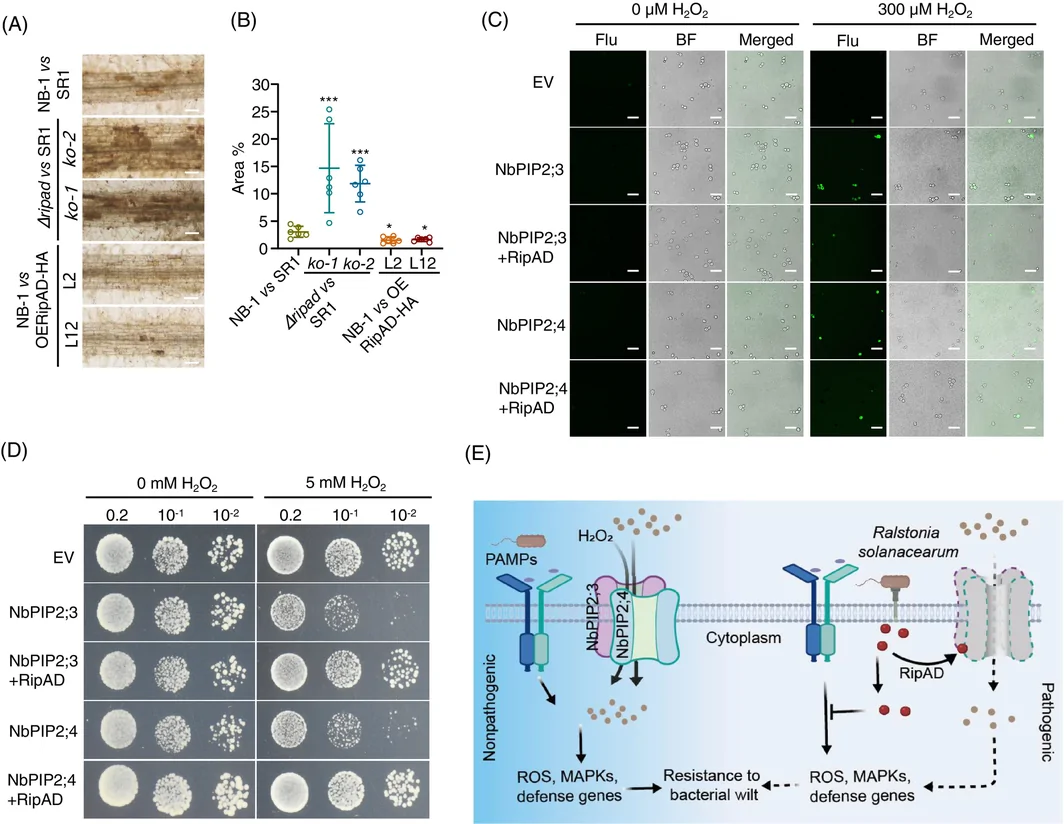
RipAD suppresses NbPIP2‐mediated H_2_O_2_ accumulation and transport. (A, B) 3,3′‐Diaminobenzidine (DAB) staining of tobacco roots inoculated with the indicated *Ralstonia solanacearum* strains. Scale bars = 100 μm. (B) Quantification of the H_2_O_2_‐stained area in roots. Data are presented as the mean ± SD, *n* = 6 root sections. Statistical significance was determined by Student's *t*‐test. Asterisks indicate significant differences compared with wild‐type control; *P < 0.05, ***P < 0.001. (C) Representative H_2_DCF‐DA fluorescence images of yeast cells expressing the indicated constructs after treatment with 0 or 300 μM H_2_O_2_. Scale bars = 50 μm. (D) Growth of the indicated yeast transformants on SG/‐Ura medium containing 0 or 5 mM H_2_O_2_. Photographs were taken after 3 days post‐incubation. (E) A schematic model illustrating that effector RipAD perturbs NbPIP oligomeric status to suppress plant immunity. Upon perception of *Ralstonia solanacearum* pathogen‐associated molecular patterns (PAMPs), the corresponding pattern recognition receptor (PRR) complex on the membrane is activated. This recognition triggers an intracellular signaling cascade. Subsequently, the membrane proteins NbPIP2;3 and NbPIP2;4 form homo‐ or hetero‐tetramers that facilitate the transport of H_2_O_2_. This oxidative burst contributes to PAMP‐triggered immunity (PTI), ultimately resulting in resistance to bacterial wilt. In the pathogenic interaction, the effector RipAD is secreted into the plant cell via the bacterial type III secretion system. RipAD translocates to the cell membrane, where it perturbs NbPIP oligomeric status. Consequently, the critical early H_2_O_2_ accumulation mediated by NbPIP oligomerization is compromised, leading to plant susceptibility to bacterial wilt.

To further determine whether RipAD interferes with NbPIP2‐mediated H_2_O_2_ transport, we employed a yeast‐based H_2_O_2_ transport activity assay. After treatment with exogenous H_2_O_2_, yeast cells expressing NbPIP2;3 or NbPIP2;4 showed stronger intracellular H_2_DCF‐DA fluorescence than the EV control, suggesting that these AQPs promote H_2_O_2_ transport in yeast cells. In contrast, co‐expression of RipAD markedly reduced the NbPIP2;3‐ and NbPIP2;4‐associated H_2_DCF‐DA fluorescence signals (Figure 7C). These data suggest that RipAD suppresses NbPIP2‐mediated H_2_O_2_ transport. We further assessed the physiological consequence of this effect using a yeast H_2_O_2_ sensitivity assay. Expression of NbPIP2;3 or NbPIP2;4 increased yeast sensitivity to H_2_O_2_, as indicated by impaired growth on medium containing 5 mM H_2_O_2_. Notably, co‐expression of RipAD alleviated the growth inhibition caused by H_2_O_2_ in yeast expressing NbPIP2;3 or NbPIP2;4 (Figure 7D). Together, these results provide functional evidence that RipAD inhibits NbPIP2;3‐ and NbPIP2;4‐mediated H_2_O_2_ accumulation and transport.

## DISCUSSION

To overcome host defense responses, pathogens employ a repertoire of effectors that suppress plant innate immunity and promote virulence. In this study, we identified RipAD from the *R. solanacearum* strain NB‐1 as a core effector that significantly contributes to virulence. RipAD is widely conserved across the RSSC and shares homology with proteins in other phytopathogens, including *Acidovorax avenae* subsp. *citrulli*, *Xanthomonas axonopodis* pv. *citrulli*, *X. campestris* pv. *vesicatoria*, and *X. oryzae* pv. *oryzae* (Jiang et al., 2022; Mukaihara et al., 2010). The molecular functions of this effector across these diverse bacterial species remain largely unknown. Specifically, AopV from *A. citrulli* strain Aac5 has been functionally characterized, which significantly suppresses ROS accumulation and PTI marker gene expression, thereby enhancing the growth of *Pseudomonas syringae* D36E in *N. benthamiana* (Jiang et al., 2022). A previous study on the phylotype I strain GMI1000 showed that RipAD suppresses flg22‐triggered immunity (Jeon et al., 2020). These findings hint at the biological significance of RipAD, but its specific biochemical mechanisms and host targets have not yet been elucidated.

Elucidating the molecular mechanisms of T3Es that lack predictable enzymatic domains remains a challenge in the study of bacterial pathogenicity. Among these proteins, the immune suppression mechanisms of several effectors with distinct subcellular localizations have been resolved. For instance, RipAB targets the nucleus to inhibit TGA transcription factors and disrupt salicylic acid signaling, whereas RipAO was found to localize to the cytoskeleton, where it disrupts microtubule networks (Jeon et al., 2025; Qi et al., 2022). Through the investigation of RipAD subcellular dynamics, we demonstrated that RipAD similarly lacks known functional domains but possesses a conserved S‐palmitoylation site at C80 and an NLS, which direct RipAD to the PM and the nucleus, respectively (Jeon et al., 2020). Mutations in the C80 and NLS sequences resulted in the loss of RipAD PM and nuclear localizations, respectively, as well as the loss of its suppression of plant PTI responses. Therefore, our data indicate that this dual localization is a prerequisite for the ability of RipAD to suppress early PTI responses. Interestingly, this unique membrane‐nuclear partitioning contrasts with a previous report on the GMI1000 strain, in which RipAD was observed in chloroplasts (Jeon et al., 2020). We further found that RipAD‐GFP unexpectedly accumulated in chloroplasts when the PM and NLSs were disrupted. This discrepancy may reflect the functional plasticity or evolutionary divergence of RipAD within the RSSC, suggesting that homologous effectors may adapt to target different host organelles to achieve pathogenicity in specific host–pathogen contexts.

AQPs are evolutionarily conserved proteins ubiquitous across living organisms, where they play vital roles in physiological processes, stress responses, and notably, plant immunity (Maurel et al., 2015; Quigley et al., 2002; Tanghe et al., 2006). Previous studies have shown that OsPIP2;2 positively regulates plant innate immunity and contributes to rice resistance against bacterial blight, leaf streak, and rice blast (Zhang et al., 2022). Similarly, NbPIP2;4 has been reported to positively regulate ROS accumulation and plant immunity (Yubo et al., 2023). In this study, we demonstrated that NbPIP2;3 and NbPIP2;4 are essential for the activation of PTI. Specifically, silencing these genes significantly suppressed multiple layers of immune responses, including csp22‐ and flg22‐induced ROS accumulation, MAPK activation, and the transcriptional induction of key defense marker genes such as *NbPti5* and *NbCYP71D20*. Additionally, plants with silenced *NbPIP2;3* and *NbPIP2;4* exhibited enhanced susceptibility to the *R. solanacearum* strain NB‐1. These results highlight the critical role of NbPIP2;3 and NbPIP2;4 as positive regulators of plant immunity against bacterial wilt.

Functional AQPs typically assemble into tetramers via homo‐ or hetero‐oligomerization, a structural prerequisite for their stability and channel activity (Barone et al., 1998; Berny et al., 2016). Here, we confirmed that NbPIP2;3 and NbPIP2;4 form both homo‐ and hetero‐oligomers. Notably, RipAD inhibited the oligomerization of NbPIP2;3 and NbPIP2;4, thereby perturbing these assemblies. This specific interference with protein–protein stoichiometry represents a sophisticated virulence mechanism that is relatively rare among bacterial effectors (Figure 7E). A classic example of such structural interference is AvrPtoB from *P. syringae*, which can physically bind to the PRR kinase BAK1, thereby disrupting the formation of the FLS2‐BAK1 immune complex (Lu et al., 2010). We speculate that RipAD discriminates PIP isoforms via sequence variations in their N termini to exert its virulence function specifically against NbPIP2;3 and NbPIP2;4. Consistent with this molecular interference phenotype, our *in planta* ROS assays showed that RipAD is associated with reduced H_2_O_2_ accumulation during *R. solanacearum* infection. Together with the yeast‐based H_2_O_2_ assays, these results support a model in which RipAD attenuates NbPIP2;3‐ and NbPIP2;4‐associated H_2_O_2_ accumulation and thereby dampens ROS signaling and downstream PTI responses. Since H_2_O_2_ acts as a core second messenger during PTI and drives defense‐related cell wall reinforcement, inhibition of NbPIP‐dependent H_2_O_2_ transport provides a plausible mechanism whereby RipAD dampens plant innate immunity and facilitates *R. solanacearum* infection. Collectively, our findings reveal a novel virulence strategy wherein *R. solanacearum* employs RipAD to dismantle the oligomeric architecture of host AQPs, thereby subverting plant immune responses to facilitate infection.

## MATERIALS AND METHODS

### Plant growth conditions and bacterial strains

*Nicotiana benthamiana* and *N. tabacum* cultivar cv. SR1 plants were grown in standardized soil at 22°C and 45% relative humidity under a 12‐h light/12‐h dark cycle. *Ralstonia solanacearum* strain NB‐1 was originally isolated from tobacco stems in China. The *RipAD* mutants (*Δripad‐1* and *Δripad‐2*) were grown on casamino acid–peptone–glucose (CPG) solid medium and cultured at 28°C in CPG liquid medium. *Agrobacterium tumefaciens* strain GV3101, harboring various constructs, was cultured in Luria‐Bertani (LB) medium supplemented with 25 μg ml^−1^ gentamicin and other appropriate antibiotics at 28°C.

### Generation of plasmid constructs

The *RipAD* coding sequence was amplified from *R. solanacearum* NB‐1 genomic DNA using primers (Table S2) incorporating *Bam*HI and *Stu*I restriction sites at the 5′ and 3′ ends, respectively. The resulting fragment was ligated into the plant protoplast expression vector pHBT under the control of the *Cauliflower mosaic virus* (*CaMV*) *35S* promoter, featuring a C‐terminal HA tag. The point mutations of *RipAD* ^*C80A*^ and *RipAD* ^*nls*^ were generated by site‐directed mutagenesis method using *pHBT‐RipAD‐HA* as the template.

The coding sequences of the PIP family members (*NbPIP2;3*, *NbPIP2;4*, *NbPIP2;9*, and *NbPIP1;3*) were amplified from *N. benthamiana* cDNA. These fragments were cloned into the pCAMBIA2300‐MYC and pCAMBIA2300‐FLAG vectors via *Bam*HI and *Stu*I digestion. For transient expression in tobacco, *RipAD*, *RipAD* ^*C80A*^, and *RipAD* ^*nls*^ were subcloned into the binary expression vector *pTF101‐GFP, pTF101‐HA*, or *pCAMBIA2300‐TurboID‐HA* for co‐localization, Co‐IP, and proximity labeling assays, respectively.

For tobacco rattle virus (TRV)‐based VIGS of *NbPIP2;3* and *NbPIP2;4*, specific fragments of 200–300 bp for each gene were identified using the VIGS Tool (https://vigs.solgenomics.net/). These fragments were amplified from *N. benthamiana* cDNA and inserted into the pTRV2 vector between the *Kpn*I and *Eco*RI restriction sites.

### Generation of *R. solanacearum* knockout, complementary and overexpression strains

To generate RipAD knockout mutants, the effector coding sequence was replaced by a kanamycin (Kan) resistance cassette via homologous recombination. Briefly, the upstream (Up) and downstream (Down) flanking regions of the target effector were amplified by PCR and ligated into the *pSK‐kana* vector along with the kanamycin resistance gene fragment to generate the recombinant construct *pSK‐Up‐Kan‐Down*. The resulting fusion fragment (*Up‐Kan‐Down*) was introduced into the NB‐1 strain via electroporation. Putative transformants were selected on CPG medium supplemented with 25 μg ml^−1^ Kan and further confirmed by PCR using effector‐specific primers (Table S2). To obtain the complementation strain, the *RipAD* coding sequence, including its native promoter, was amplified from *R. solanacearum* NB‐1 genomic DNA and cloned into the *pBBR1‐HA* vector to generate *pBBR1‐ProRipAD‐RipAD‐HA* vector. This construct was then transformed into the *∆ripad* mutants by electroporation. Complementation strains were selected using both kanamycin and gentamicin and subsequently validated by PCR using *RipAD* cloning primers and immunoblotting with an anti‐HA antibody.

### Assays for bacterial biological characteristics

To monitor bacterial growth, strains were cultured overnight at 28°C in CPG liquid medium. Cells were harvested, resuspended in fresh CPG medium, and adjusted to an initial OD_600_ = 0.05. The OD_600_ values were recorded every 8 h to generate growth curves.

Biofilm formation was assessed using the crystal violet staining method. Bacterial cells were adjusted to OD_600_ = 0.1 in CPG medium and inoculated into 96‐well polystyrene plates. After incubation at 28°C for 48 h, the biofilms were stained with 0.1% (w/v) crystal violet for 15–20 min, followed by dissolution in 95% ethanol for 30 min. Biofilm capacity was quantified by measuring the absorbance at 590 nm.

For EPS production analysis, bacterial cultures were adjusted to OD_600_ = 0.1 and incubated in a shaker at 28°C for 72 h. The supernatant was collected and treated with acetylacetone‐Na_2_CO_3_ and *p*‐dimethylaminobenzaldehyde (1.5%, w/v). The absorbance at 525 nm was measured to evaluate EPS production capacity.

Cellulase secretion was measured on carboxymethyl cellulose‐sodium (CMC‐Na) agar plates. A 5‐mm diameter hole was punched in the center of the CMC‐Na plate, into which 30 μl of bacterial suspension (OD_600_ = 1.0) was added. The plates were incubated at 28°C for 18 h, then stained with 0.2% (w/v) Congo red for 30 min, and decolored with 1 mol L^−1^ NaCl solution three times. The diameter of the degradation halo was measured to assess cellulase activity.

### Bacterial infection assays

For *R. solanacearum* soil‐drenching inoculation, 8–12 tobacco plants per genotype (4–5 weeks old) were used. The root systems were slightly wounded by inserting a knife into the soil three times prior to inoculation. Each plant was then inoculated with 10 ml of *R. solanacearum* suspension adjusted to 10^8^ colony‐forming units per ml (CFU ml^−1^) in sterile ddH_2_O. Inoculated plants were maintained in a growth chamber at 28°C and 75% relative humidity under a 12‐h light/12‐h dark photoperiod. The plants were watered on alternating dpi. Disease symptoms were rated daily on a scale of 0–4 (0: no symptoms; 1: 1–25% of leaves wilted; 2: 26–50% wilted; 3: 51–75% wilted; and 4: 76–100% wilted or dead). For survival rate analysis, a disease index ≤2 was defined as survival, while a disease index >2 was recorded as death. Bacterial populations in the stem were determined at 7 dpi. Stem sections (approximately 1–2 cm above the soil line) were harvested, weighed, and surface‐sterilized with 70% ethanol for 30 sec. The samples were then homogenized in sterile ddH_2_O. Serial dilutions of the homogenates were spread on solid CPG medium, and CFUs were counted after incubation at 28°C for 48 h. Bacterial density was expressed as log_10_CFU g^−1^ fresh weight.

### VIGS assay

*Agrobacterium tumefaciens* strain GV3101, harboring the TRV constructs (pTRV1 and pTRV2), was cultured for the infiltration of *N. benthamiana*. *Agrobacterium* cells carrying TRV‐RNA1 were adjusted to OD_600_ = 1.5 and mixed with an equal volume of cells carrying various TRV‐RNA2 constructs, including TRV‐GFP (control), TRV‐NbPIP2;3, or TRV‐NbPIP2;4. The cotyledons of 2‐week‐old *N. benthamiana* plants were infiltrated with the *Agrobacterium* mixtures using a needleless syringe. The silenced plants were maintained in a growth chamber as described above. The silencing efficiency was assessed by RT‐qPCR analysis at 2 weeks post‐infiltration. Subsequently, *R. solanacearum* inoculation was performed on the silenced plants to evaluate their susceptibility to bacterial wilt.

### Transformation of tobacco plants

The recombinant vector *pCAMBIA2300‐RipAD‐HA* was transformed into *A. tumefaciens* strain GV3101 and introduced into *N. tabacum* cultivar cv. SR1 using the leaf disk method, as described previously with minor modifications (Lv, Chen, et al., 2021; Lv, Dai, et al., 2021). Briefly, *A. tumefaciens* GV3101 carrying the construct was cultured in LB medium containing kanamycin and gentamicin at 28°C until the culture reached an OD_600_ of 0.6–0.8. The bacterial cells were collected by centrifugation and resuspended in MS medium supplemented with 30 g L^−1^ sucrose and 20 mg L^−1^ acetosyringone. Tobacco leaf disks were immersed in the bacterial suspension for 8–10 min, co‐cultivated on antibiotic‐free MS medium in the dark for 3 days, and then transferred to differentiation medium containing MS salts, 2.5 mg L^−1^ 6‐benzylaminopurine, 0.2 mg L^−1^ NAA, 50 mg L^−1^ kanamycin, and 150 mg L^−1^ timentin to promote shoot regeneration. Regenerated shoots were transferred to rooting medium containing MS salts, 0.1 mg L^−1^ NAA, and 200 mg L^−1^ cefotaxime. Resistant plantlets were transplanted into soil for further analysis.

### Subcellular localization analysis

The binary constructs *pTF101‐GFP*, *pTF101‐RipAD‐GFP*, *pTF101‐RipAD* ^*C80A*^ *‐GFP*, and *pTF101‐RipAD* ^*nls*^ *‐GFP* were individually transformed into *A. tumefaciens* strain GV3101. Leaves of 4‐week‐old *N. benthamiana* were infiltrated with the *Agrobacterium* cultures adjusted to a final OD_600_ = 1.5. Confocal micrographs were acquired at 2 dpi using a Leica SP8 confocal laser scanning microscope. The excitation wavelengths were set at 488 nm for GFP and 633 nm for chlorophyll autofluorescence. Images were captured and processed using the Leica LAS X software.

For co‐localization analysis, GFP, RipAD‐GFP, *C80A*‐GFP, or *nls*‐GFP was transiently co‐expressed with H2B‐mCherry or HIR4‐mCherry in *N. benthamiana* leaves by *Agrobacterium* infiltration. Two days after infiltration, fluorescence was visualized with Leica SP8 confocal microscope. The mCherry fluorescence was excited at 587 nm.

### ROS burst assay

To measure the ROS burst in *N. benthamiana*, RipAD was transiently expressed in *N. benthamiana* leaves with *Agrobacterium*‐mediated infiltration. At 2 dpi, 24 leaf disks (0.25 cm^2^) were harvested, cut into strips, and incubated overnight in sterile ddH_2_O in 96‐well plates to eliminate the wounding effect. The water was then replaced with a reaction solution containing 50 μM L‐012, 10 μg ml^−1^ horseradish peroxidase (HRP), and either 100 nM flg22 or 100 nM csp22. Luminescence was recorded immediately using a multimode microplate reader (Tecan Spark 10M) over a 45‐min period. ROS production was quantified as RLU, and data were presented as the mean of multiple replicates.

### MAP kinase assay

The leaf disks (0.25 cm^2^) were obtained from the *N. benthamiana* 2 days post‐infiltration and transferred to the 12‐well plate with ddH_2_O for overnight recovery. Each sample contained two leaf disks and was treated with 100 nM flg22 or 100 nM csp22 for 0, 5, 15, and 30 min. Following treatment, samples were immediately frozen in liquid nitrogen and ground in sodium dodecyl sulfate (SDS) loading buffer, followed by boiling at 95°C for 10 min. Total proteins were separated by 10% SDS‐polyacrylamide gel electrophoresis (SDS‐PAGE). The expression of effector proteins and the phosphorylation of MAPKs were detected by immunoblotting using anti‐HA and anti‐phospho‐p44/42 MAPK (anti‐pERK1/2) antibodies, respectively.

### Gene expression and RT‐qPCR analysis

Total RNA was extracted from *N. benthamiana* leaves expressing either the EV or RipAD following treatment with 100 nM csp22 for 0, 1, and 3 h. For each sample, 1 μg of total RNA was reverse‐transcribed to synthesize first‐strand cDNA using a reverse transcription kit. RT‐qPCR was performed using SYBR Green SuperMix. The relative expression levels of defense‐related genes were normalized to the internal control gene *NbEF‐1α* using the 2^−ΔΔCt^ method.

### Large‐scale immunoprecipitation and LC‐MS/MS analysis

*Nicotiana benthamiana* leaf tissues were harvested 2 days post‐infiltration with *Agrobacterium* strains carrying RipAD‐GFP or RipAD‐TurboID‐HA and immediately frozen in liquid nitrogen. Tissues were lysed in Co‐IP buffer (20 mM Tris‐HCl, pH 7.5; 100 mM NaCl; 1 mM EDTA; 2 mM DTT; 10% glycerol; 0.5% Triton X‐100; and 1× protease inhibitor cocktail). After centrifugation at 12 000 × g for 15 min at 4°C, the supernatant was incubated with 20 μl GFP‐Trap agarose beads for 3 h at 4°C. The beads were subsequently washed three times with washing buffer (20 mM Tris‐HCl, pH 7.5; 100 mM NaCl; 1 mM EDTA; and 0.1% Triton X‐100). The immunoprecipitated proteins were separated by SDS‐PAGE and subjected to LC‐MS/MS analysis at Applied Protein Technology (Shanghai, China).

### Firefly split‐luciferase complementation assay

For split‐LUC assays, *A. tumefaciens* harboring *pCAMBIA1300‐RipAD‐cLUC* was co‐infiltrated with strains carrying nLUC‐tagged NbPIPs (*NbPIP2;3‐nLUC*, *NbPIP2;4‐nLUC*, *NbPIP2;9‐nLUC*, or *NbPIP1;3‐nLUC*) into *N. benthamiana* leaves. At 2 days post‐infiltration, the infiltrated areas were treated with 1 mM d‐luciferin and kept in the dark for 5 min. Bioluminescence signals were captured using a charge‐coupled device (CCD) camera (Tanon 5200 Multi). To quantify luciferase activity, leaf disks (10 mm diameter) were incubated with 1 mM luciferin in a 96‐well plate, and luminescence intensity was recorded using a multimode microplate reader (Tecan Spark 10M).

### DAB staining for H_2_O_2_ contents in roots

DAB staining was performed as previously described with slight modifications (Li et al., 2025). A 0.04% (w/v) DAB solution (Aladdin, D106468, Shanghai, China) containing 0.05% (v/v) Tween 20 was freshly prepared. To facilitate DAB dissolution, HCl was added and stirred until the DAB dissolved, and the pH was adjusted to 5.0 using a phosphate buffer. Tobacco roots were collected after *R. solanacearum* inoculation and soaked in DAB solution for 3 h in the dark. The dark brown roots were directly observed under a light microscope without decolorization (Leica, DM2500, Wetzlar, Germany). Whole roots were first scanned under the microscope, and images of root regions showing visible DAB staining were captured under identical imaging conditions. The DAB‐positive area and total root area in each image were quantified using ImageJ software with the same threshold parameters. Relative H_2_O_2_ accumulation was calculated as the ratio of the DAB‐positive area to the total root area in the same visual field, and the resulting area percentage was used for statistical comparisons among groups.

### H_2_O_2_ transport assay

According to the reported method (Tian et al., 2016), yeast (*Saccharomyces cerevisiae*) strain BY4741 (*MATa his3Δ1 leu2Δ0 met15Δ0 ura3Δ0*) and the pESC‐URA vector (Honorgene, Changsha, China) were used in this study. Yeast cells were cultured in YPDA medium. The coding sequences of *NbPIP2;3* and *NbPIP2;4* were amplified from *N. benthamiana* cDNA using the primers listed in Table S2. These fragments were cloned into the pESC‐URA vector via *Bam*HI and *Sma*I digestion. The *RipAD* coding sequence was amplified from *R. solanacearum* NB‐1 genomic DNA and cloned into the pESC‐URA‐NbPIP2 constructs via *Eco*RI/*Spe*I or *Eco*RI/*Not*I digestion. Yeast transformants were cultured overnight in SD/‐Ura medium at 28°C. The cells were collected by centrifugation, washed twice with sterile H_2_O, resuspended in sterile H_2_O, and transferred into SG/‐Ura medium to induce gene expression. When the culture reached an OD_600_ of approximately 1.0, yeast cells were treated with 300 μM H_2_O_2_ for 45 min at room temperature. After H_2_O_2_ treatment, the cells were stained with 10 μM H_2_DCF‐DA for 20 min at room temperature in the dark. The fluorescence of oxidized DCF was observed using a fluorescence microscope with excitation at 488 nm and emission at approximately 525–530 nm.

For the yeast growth inhibition assay, induced yeast cells were adjusted to OD_600_ = 0.2 and then serially diluted 10‐fold to OD_600_ = 0.02 and 0.002. Then, 8 μl of each dilution was spotted onto SG/‐Ura medium with or without 5 mM H_2_O_2_. Photographs were taken after incubation on the plates for 3 days at 28°C.

### Quantification and statistical analysis

Statistical analyses were performed using GraphPad Prism 9.0. Data are presented as mean ± standard deviation or mean ± standard error of the mean from independent biological replicates. Significant differences between treatments were determined using Student's *t*‐test or one‐way anova as specified in the corresponding figure legends (*P* < 0.05). The statistical analysis methods are described in the figure legends.

## ACCESSION NUMBERS

The following phenotypic, genotypic, and functional terms are of significance to the work described in this paper: NbPIP2;3 Gramene: Nbe14g05380; NbPIP2;4 Gramene: Nbe04g03590; NbPIP2;6 Gramene: Nbe10g00570; NbPIP1;3 Gramene: Nbe06g29240.

## AUTHOR CONTRIBUTIONS

BN performed the experiments and organized the figures. SY, YL, YZ, and PL assisted with the data collation and discussion. JX, YL, YF, JC, DJ, and XY analyzed the data and provided critical feedback. BL supervised the work, designed the experiments, and wrote the manuscript with input from all coauthors.

## CONFLICT OF INTEREST

The authors declare no competing interests.

## Supporting information


**Figure S1.** RipAD is not required for basal bacterial growth and extracellular virulence factor production *in vitro*.
**Figure S2.** Evolutionary conservation of RipAD and its orthologs in phytopathogenic bacteria.
**Figure S3.** Structural characterization and functional verification of RipAD localization variants.
**Figure S4.** Identification and molecular characterization of NbPIPs as RipAD‐interacting partners.
**Figure S5.** Specificity of RipAD interference with NbPIP oligomerization.
**Figure S6.** Verification of VIGS efficiency of *NbPIP2;3*‐ and *NbPIP2;4*‐silenced plants.
**Table S2.** Primers used in this study.

**Table S1.** List of potential RipAD interacting proteins identified by TurboID and MS analysis.

## Data Availability

All data supporting the findings of this study are available in the article and the accompanying Supporting Information.
