# Supplementary material for: A Ralstonia solanacearum effector RipAD perturbs aquaporin NbPIP oligomeric status to suppress plant immunity
Source: Plant J. 2026 Oct 8;128(1):e71111. doi: 10.1111/tpj.71111 (PMC13647096; doi:10.1111/tpj.71111)
Supplement: Supplementary file 1 [file TPJ-128-0-s001.docx]

## **Supplementary Information**

**Figure S1. RipAD is not required for basal bacterial growth and extracellular virulence factor production *in vitro*.**

(A) PCR analysis confirms the successful generation of RipAD deletion mutants (*Δripad*). Genomic DNA from the wild-type NB-1 and three independent *Δripad* lines was amplified using primers specific to the *R. solanacearum* flagellin gene (*fliC*, internal control), the full-length *RipAD* gene, and the *kanamycin* resistance cassette (*RipAD* flanking regions fused with the *kanamycin* gene).

(B) Immunoblot analysis confirms the expression of RipAD in the complemented strains. Total proteins from NB-1, *Δripad*, and three independent *ComRipAD* lines (expressing HA-tagged RipAD) were analyzed using an anti-HA antibody. Coomassie brilliant blue (CBB) staining serves as a loading control.

(C) *RipAD* deletion does not alter colony morphology. Strains were grown on casamino acid-peptone-glucose (CPG) solid medium supplemented with tetrazolium chloride (TTC). Photographs were taken after 2 days of growth at 28°C. Scale bars, 0.5 cm.

(D) RipAD is dispensable for normal bacterial growth *in vitro*. Growth curves of the indicated strains in CPG liquid medium were determined by continuously monitoring the cell density at OD_600_. Data are presented as mean ± SD (n = 3); the same letters indicate no significant differences according to one-way ANOVA (*p* < 0.05).

(E) RipAD does not affect biofilm formation. Biofilm formation was assessed using the crystal violet staining method. The biofilm capacity of the indicated strains was quantified by measuring the absorbance at 590 nm.

(F) RipAD is not required for extracellular polysaccharide (EPS) production. The levels of soluble EPS from cultures grown in CPG liquid medium were quantified at OD_525_.

(G) RipAD does not influence cellulase secretion. Cellulase activity was evaluated on carboxymethyl cellulose sodium (CMC-Na) solid medium by measuring the diameter of the degradation zone.

Data in (E-G) are presented as box plots showing all individual data points (n = 8); the center line represents the median, and whiskers indicate the minimum and maximum values. 'ns' indicates not significant, as determined by Student’s two-tailed *t*-test.

**Figure S2. Evolutionary conservation of RipAD and its orthologs in phytopathogenic bacteria.**

(A) RipAD is evolutionarily related to the type III effectors XopV and AopV. Phylogenetic analysis of RipAD homologs. The tree was constructed using the Maximum Likelihood method. It groups RipAD variants from diverse *R. solanacearum* strains (phylotypes I, II, III, IV) with the orthologous effectors XopV from *Xanthomonas* spp., AopV from *Paracidovorax* (formerly *Acidovorax*) spp., and hypothetical proteins from *Rhizobium*. Bootstrap values are shown at the nodes.

(B) RipAD shares conserved sequence motifs with its orthologs. Multiple sequence alignment of RipAD isoforms (from *R. solanacearum* strains NB-1, GMI1000, CFBP2957, MolK2, CMR15) and orthologs from *A. citrulli* (AopV) and *X. oryzae* (XopV). Dark blue shading indicates high amino acid identity. The orange dashed boxes highlight highly conserved motifs, particularly in the C-terminal region.

**Figure S3. Structural characterization and functional verification of RipAD localization variants.**

(A) Schematic representation of predicted functional motifs in RipAD. The diagram highlights the N-terminal chloroplast transit peptide (cTP), the putative S-palmitoylation site at cysteine 80 (C80) responsible for PM tethering, and the NLS.

(B) Verification of protein expression for RipAD variants. *N. benthamiana* leaves were agroinfiltrated with constructs expressing free GFP, wild-type RipAD-GFP, *C80A*-GFP, or *nls*-GFP. Ponceau S staining of RBC serves as a loading control.

(C) RipAD-GFP co-localizes with the plasma membrane marker HIR4-mCherry in *N. benthamiana* leaves. GFP, RipAD-GFP, C80A-GFP, or nls-GFP was co-expressed with HIR4-mCherry in *N. benthamiana* leaves. Fluorescence signals were observed by confocal microscopy at 2 days after co-expression. Fluorescence intensity profiles were measured along the cyan lines indicated in the merged images and are shown in the right panels. Scale bars, 25 μm.

(D) RipAD-GFP co-localizes with H2B-mCherry in the nucleus. GFP, RipAD-GFP, C80A-GFP, or nls-GFP was co-expressed with H2B-mCherry in *N. benthamiana* leaves. Scale bars, 25 μm.

(E, F) PM and nuclear localizations are required for RipAD to suppress flg22-induced immune gene expression. Gene expression was normalized to the internal control *NbEF1α*. Data are presented as mean ± SD (n = 3) with individual data points displayed. Different letters indicate statistically significant differences according to one-way ANOVA (*p* < 0.05). All experiments were repeated three times with similar results.

**Figure S4. Identification and molecular characterization of NbPIPs as RipAD-interacting partners.**

(A) RipAD associates with NbPIP family members *in vivo*. Representative LC-MS/MS spectra of unique peptides corresponding to NbPIP2;9 and NbPIP1;3. These candidate proteins were identified by intersecting data from two independent approaches: Co-IP (using RipAD-GFP) and TurboID-mediated proximity labeling (using RipAD-TurboID-HA) in *N. benthamiana* leaves.

(B) Phylogenetic analysis groups the identified NbPIPs with conserved Nicotiana aquaporin subfamilies. The phylogenetic tree was constructed using the neighbor-joining method (MEGA12) based on amino acid sequences of PIPs from *N. benthamiana*, *N. sylvestris*, and *N. tomentosiformis*. The RipAD-interacting NbPIPs are highlighted in orange. Bootstrap values (%) from 1000 replicates are shown at the nodes.

(C) Predicted topological structure of NbPIPs. Schematic representation of the NbPIP protein structure, featuring six transmembrane helices (TMH1–6) and five connecting loops (Loop A–E). The N- and C-termini are located in the cytoplasm.

(D) Structural modeling of the RipAD–NbPIP interaction. Ribbon diagram showing the predicted docking interface between RipAD (blue) and the NbPIP2;3/4 complex (cyan/green). Key interacting residues are indicated in the zoomed-in view.

**Figure S5**. **Specificity of RipAD interference with NbPIP oligomerization.**

(A) NbPIP2 subfamily members, but not NbPIP1;3, form stable homodimers. Split-luciferase complementation (Split-LUC) assays were performed in *N. benthamiana* leaves.

(B) RipAD does not affect the homodimerization of NbPIP2;9. *N. benthamiana* leaves were co-infiltrated with NbPIP2;9-nLUC and NbPIP2;9-cLUC, along with increasing concentrations of RipAD-HA (OD_600_ ratios: 0, 0.1, 0.5, 1.0). Representative luminescence images (left) and quantification (right) are shown.

(C) RipAD does not interfere with the heterodimerization between NbPIP2;3 and NbPIP2;9. Split-LUC assays were performed using the NbPIP2;3-nLUC and NbPIP2;9-cLUC pair in the presence of increasing concentrations of RipAD-HA as in (B).

Data in (B) and (C) are presented as mean ± SD (n = 3). Statistical analysis was performed using one-way ANOVA followed by Tukey's test (*p* < 0.05). The letter 'a' indicates no statistically significant difference among samples.

(D) RipAD promotes the degradation of NbPIP2;3-MYC and NbPIP2;4-MYC. NbPIP2-MYC was co-expressed with empty control, RipAD-HA in *N. benthamiana* leaves. + and ++ indicate that the OD_600_ ratio of *Agrobacterium* containing RipAD-HA to that containing NbPIP2 is 0.25:1 and 0.5:1, respectively.

**Figure S6. Verification of VIGS efficiency of *NbPIP2;3*- and *NbPIP2;4*-silenced plants.**

(A) Simultaneous silencing of *NbPIP2;3* and *NbPIP2;4* does not alter plant growth or morphology. Representative photographs of *N. benthamiana* plants at 2 weeks post-infiltration with the indicated TRV constructs. *TRV:PDS* (phytoene desaturase) serves as a visual positive control for VIGS efficacy, displaying characteristic photobleaching. *TRV:GFP* serves as the negative control.

(B) VIGS treatment effectively knocks down *NbPIP2;3* and *NbPIP2;4* transcript levels. Relative expression levels were quantified by RT-qPCR using gene-specific primers. Expression was normalized to the *NbEF1α* internal control and is presented relative to the TRV:GFP control. Data are presented as mean ± SD (n = 3). Statistical significance was determined using Student’s two-tailed t-test (****p* < 0.001).

(C) Protein expression levels of RipAD-HA in the indicated lines. The wild-type tabacco SR1 was used as control.

Supplementary Table 2: Primers used in this study.

| **Gene** | **Forward Primer** | **Reverse Primer** | **Application** |
| --- | --- | --- | --- |
| RipAD | CGGGATCCATGTTGAAAATCGGCCGTATCAC | AAGGCCTCCAGTCGAGCTGCGGATC | CDS amplification |
| NbPIP2;3 | CGCGGATCCATGAGTCTATTCTTTGGTGAACAAAT | GAAGGCCTGGCATTGCTCCTGAATGAAC | CDS amplification |
| NbPIP2;4 | CGCGGATCCATGACTAAAGAAGTAGAGGTAGCAAC | GAAGGCCTGGCATTGCTCCTGAAGGAA | CDS amplification |
| NbPIP2;9 | CGCGGATCCATGTCAAAGGACGTAATTGAAGAAG | GAAGGCCTGTTGGTTGGATTGCTGCG | CDS amplification |
| NbPIP1;3 | CGCGGATCCATGGCAGAAAACAAAGAAGAAGAT | GAAGGCCTAGCCTTGCTCTTGAATGGAA | CDS amplification |
| NbPIP2;3NT | CGCGGATCCATGAGTCTATTCTTTGGTGAACAAAT | GAAGGCCTAAGAATTCCAACACCACCACA | Truncation |
| NbPIP2;3CT | CGCATGGGATCCATGGGAATTGCATGGGCATTTG | GAAGGCCTGGCATTGCTCCTGAATGAAC | Truncation |
| NbPIP2;4NT | CGCGGATCCATGACTAAAGAAGTAGAGGTAGCAAC | GAAGGCCTAAGAATACCAACGCCGCCA | Truncation |
| NbPIP2;4CT | CGCATGGGATCCATGGGTATTGCTTGGGCTTTTGG | GAAGGCCTGGCATTGCTCCTGAAGGAA | Truncation |
| NbPIP2;3 | CGCGGATCCATGAGTCTATTCTTTGGTGAACAAAT | GAAGGCCTTTAGGCATTGCTCCTGAATGAAC | cLUC |
| NbPIP2;4 | CGCGGATCCATGACTAAAGAAGTAGAGGTAGCAAC | GAAGGCCTTTAGGCATTGCTCCTGAAGGAA | cLUC |
| NbPIP2;9 | CGCGGATCCATGTCAAAGGACGTAATTGAAGAAG | GAAGGCCTTCAGTTGGTTGGATTGCTGCG | cLUC |
| NbPIP1;3 | CGCGGATCCATGGCAGAAAACAAAGAAGAAGAT | GAAGGCCTTTAAGCCTTGCTCTTGAATGGAA | cLUC |
| RipAD | CGGGATCCATGTTGAAAATCGGCCGTATCAC | AAGGCCTTCACCAGTCGAGCTGCGGATC | cLUC |
| RipAD^C80A^ | GAGCGCACGCTGTCCGCTGCCGGGTCGCTGCAG |  | Site  mutagenesis |
| RipAD^nls^ | CAGCCGGCGCGCGCCGCGGCCGCCGCTGCTGCGATCCAGCCCGAT |  | Site  mutagenesis |
| NbPIP2;3 | CGCGGATCCATGAGTCTATTCTTTGGTGAACAAAT | TCCCCCGGGGGCATTGCTCCTGAATGAAC | pESC-URA |
| NbPIP2;4 | CGCGGATCCATGACTAAAGAAGTAGAGGTAGCAAC | TCCCCCGGGGGCATTGCTCCTGAAGGAA | pESC-URA |
| RipAD | CGGAATTCATGTTGAAAATCGGCCGTATCAC | CGGACTAGTCCAGTCGAGCTGCGGATC | pESC-URA-NbPIP2;3 |
| RipAD | TTCAACCCTCACTAAAGGGCGGCCGCATGTTGAAAATCGGCCGTATCAC | TCCTTGTAATCCATCGATACTAGTCCAGTCGAGCTGCGGATC | pESC-URA-NbPIP2;4 |
| RipAD-up | GGTACCGAAGGCCGCAACCGGTAC | GAATTCACCTGCTTGTTGCGGAGG | Deletion |
| RipAD-dn | ACTAGTGACTGGTGACGGGCGATC | GAGCTCTCGCCTGATGCGAGACAACC | Deletion |
| RipAD-Pro | TAAAGGGAACAAAAGCTGGGTACCGAAGGCCGCAACCGGTAC | TGGAACGTCGTATGGGTATCTAGACCAGTCGAGCTGCGGATC | Complement |
| NbPIP2;3 | CGCGGATCCTTTTACAGAGCCATAATAGCTGAAT | GGGAATTCGGCTCCTAAACACTGAGC | VIGS |
| NbPIP2;4 | CGGGGTACCTTTCATTCTTGTTTACTGCACCG | TCCCCCGGGGGGTCAGTAGCAGAGAAGAC | VIGS |
| NbPIP2;3 | TTTTTGCTGCTACTGATCCTAA | GGGTTAATACCAGTTCCAGT | RT-qPCR |
| NbPIP2;4 | CCTCCACCAGCTCCTTTAG | CTTGCCAAAAACAGCCCAAAT | RT-qPCR |
| NbEF1a | CACGCATTGCTTGCTTTCA | TCCATCTTGTTACAGCAGCAAATC | RT-qPCR |
| NbCYP71D20 | AAGGTCCACCGCACCATGTCCTTAGAG | AAGAATTCCTTGCCCCTTGAGTACTTGC | RT-qPCR |
| NbNbPti5 | CCTCCAAGTTTGAGCTCGGATAGT | CCAAGAAATTCTCCATGCACTCTGTC | RT-qPCR |
| NbAcre31 | AATTCGGCCATCGTGATCTTGGTC | GAGAAACTGGGATTGCCTGAAGGA | RT-qPCR |
| KAN | AAGGAAGCGGAACACGTAGA | AAGGATTTGCAGACTACGGG | CDS amplification |
| Rsol-fliC | GAACGCCAACGGTGCGAACT | GGCGGCCTTCAGGGAGGTC | Identification |
